# Supplementary material for: Underweight predicts extubation failure after planned extubation in intensive care units
Source: PLoS One. 2023 Apr 13;18(4):e0284564. doi: 10.1371/journal.pone.0284564 (PMC10101394; doi:10.1371/journal.pone.0284564)
Supplement: Attachment — Submitted filename: Response.docx [file pone.0284564.s003.docx]

**Responses to Reviewer’s Comments**

**Reviewer #2**:

Here you receive my 2nd review of the manuscript entitled “Underweight Predicts Extubation Failure after Planned Extubation in Intensive Care Units “with short title: “Underweight and extubation failure“ and ID: PONE-D-22-30747R1.
The authors took our previous comments very seriously in trying to improve understanding of their study results. However, I have some questions. When you answer one of our questions regarding lower body mass, vs overweight you found that besides underweight other factors more related total ventilation were associated with extubation failure, e.g. decreased maximal inspiratory airway pressure (aOR, 1.05 per 1-cmH2O decrease; 95% CI 1.00-1.09) and having ESRD. Did you find any association between relative underweight and decrease of maximal inspiration pressure and/or ESRD. Ans also in other words, did patients with underweight within the population and no ESRD and normal maximal inspiration pressure also show an increased risk of extubation failure?

**Response:**

Thank you for the query. To exam the association and potential multicollinearity between underweight, having ESRD and having a low MIP level, we calculated the variance inflation factor (VIF) of the final multivariate logistic model and no potential multicollinearity was detected.

| Variables | Variance Inflation Factor |
| --- | --- |
| Underweight vs. normoweight | 1.29 |
| Overweight vs. normoweight | 1.66 |
| Maximal inspiratory pressure | 2.51 |
| Smoking | 1.52 |
| Having ESRD | 1.09 |

Furthermore, as an additional sensitivity analysis, we excluded those with ESRD (n=20) and those with a MIP<20 cmH_2_O (n=19). In the remaining 229 patients, 105 were normoweight and the proportion of extubation failure was 3.8% (4/105) while the proportion of extubation failure were 12.8% (5/39) and 2.7% (2/74) for underweight and overweight patients, respectively. In the univariate logistic regression, being underweight remained borderline associated with extubation failure (OR compared to normoweight patients, 3.37; 95% CI, 0.86-13.2; p=0.08). This additional analysis were added in the result section.

In my opinion the results are presented in a better understandable way and also their conclusions are improved, by making possible other confounders more visible and proving more weight to the the “underweight” aspects of their study, such as for instance ” We have changed the multivariate analysis and treated the BMI as categorical variables. In the new analysis, being underweight (using the WHO definition), is associated with an 3.80 increase of risk of extubation failure when compared to those with normal weight (95% CI, 1.23-11.7). “
For instance, also table 3 now provides better valuable information supporting the conclusion.
**Response:**

Thank you for the comments and helping us improving the manuscript.

Minor remarks
P6 “…and a lower Glasgow come scale …“ change come into coma
P11 “…succeed on their second attempt” change succeed into past tense succeed
P16 “…while being underweight was had borderline association (OR compared to normoweight patients, 2.67; 95% CI, 0.97-7.35)” remove here the word “was”
Ad Table 3 please provide in a legend or other remark what is meant with the asterisk * within the table?
P19 post-operative may be written as postoperative
P23 “…might differ across among different…”change sentence into “Finally, since the weight distribution may differ between different countries or ethnicities, our results should be generalized with caution between different population groups.”

**Response:**

Thank you for the correction. The text was amended accordingly.

In the reference

For instance Ref 25 = Ref 35, please check total list for possible doubles and the right presentation?

Moreover, this reference is not numbered! “Nemer SN, Barbas CS, Caldeira JB, Guimarães B, Azeredo LM, Gago R, et al. Evaluation of maximal inspiratory pressure, tracheal airway occlusion pressure, and its ratio in the weaning outcome. J Crit Care. 2009;24(3):441-6.”

**Response:**

Thank you for the correction. We have rechecked the order and listing of references.
